# Impact of timing of renal replacement therapy initiation on outcome of septic acute kidney injury

**DOI:** 10.1186/cc10252

**Published:** 2011-06-06

**Authors:** Yu-Hsiang Chou, Tao-Min Huang, Vin-Cent Wu, Cheng-Yi Wang, Chih-Chung Shiao, Chun-Fu Lai, Hung-Bin Tsai, Chia-Ter Chao, Guang-Huar Young, Wei-Jei Wang, Tze-Wah Kao, Shuei-Liong Lin, Yin-Yi Han, Anne Chou, Tzu-Hsin Lin, Ya-Wen Yang, Yung-Ming Chen, Pi-Ru Tsai, Yu-Feng Lin, Jenq-Wen Huang, Wen-Chih Chiang, Nai-Kuan Chou, Wen-Je Ko, Kwan-Dun Wu, Tun-Jun Tsai

**Affiliations:** 1 Division of Nephrology, Department of Internal Medicine, National Taiwan University Hospital, 7 Chung-Shan South Road, Taipei 100, Taiwan; 2 Division of Nephrology, Department of Internal Medicine, National Taiwan University Hospital, Yun-Lin branch, 579, Sec. 2, Yunlin Rd., Douliu City, Yunlin County 640, Taiwan; 3 Department of Internal Medicine, Cardinal Tien Hospital, 362, Zhongzheng Rd., Xindian City, Taipei County 231, Taiwan; 4 Division of Nephrology, Department of Internal Medicine, Saint Mary's Hospital, 160 Chong-Cheng South Road, Lotung 265, I-Lan, Taiwan; 5 Department of Internal Medicine, Buddhist Dalin Tzu Chi General Hospital, 2 Minsheng Road, Dalin Township, Chiayi County 622, Taiwan; 6 Department of Surgery, National Taiwan University Hospital, 7 Chung-Shan South Road, Taipei 100, Taiwan; 7 Department of Internal Medicine, Tao-Yuan General Hospital, 6 Sinfu 2nd Road, Sinwu Township, Taoyuan County 327, Taiwan; 8 Department of Traumatology, National Taiwan University Hospital, 7 Chung-Shan South Road, Taipei 100, Taiwan; 9 National Taiwan University Hospital Study Group on Acute Renal Failure, National Taiwan University Hospital, No.7, Chung Shan S. Rd, Taipei 100, Taiwan

## Abstract

**Introduction:**

Sepsis is the leading cause of acute kidney injury (AKI) in critical patients. The optimal timing of initiating renal replacement therapy (RRT) in septic AKI patients remains controversial. The objective of this study is to determine the impact of early or late initiation of RRT, as defined using the simplified RIFLE (risk, injury, failure, loss of kidney function, and end-stage renal failure) classification (sRIFLE), on hospital mortality among septic AKI patients.

**Methods:**

Patient with sepsis and AKI requiring RRT in surgical intensive care units were enrolled between January 2002 and October 2009. The patients were divided into early (sRIFLE-0 or -Risk) or late (sRIFLE-Injury or -Failure) initiation of RRT by sRIFLE criteria. Cox proportional hazard ratios for in hospital mortality were determined to assess the impact of timing of RRT.

**Results:**

Among the 370 patients, 192 (51.9%) underwent early RRT and 259 (70.0%) died during hospitalization. The mortality rate in early and late RRT groups were 70.8% and 69.7% respectively (*P *> 0.05). Early dialysis did not relate to hospital mortality by Cox proportional hazard model (*P *> 0.05). Patients with heart failure, male gender, higher admission creatinine, and operation were more likely to be in the late RRT group. Cox proportional hazard model, after adjustment with propensity score including all patients based on the probability of late RRT, showed early dialysis was not related to hospital mortality. Further model matched patients by 1:1 fashion according to each patient's propensity to late RRT showed no differences in hospital mortality according to head-to-head comparison of demographic data (*P *> 0.05).

**Conclusions:**

Use of sRIFLE classification as a marker poorly predicted the benefits of early or late RRT in the context of septic AKI. In the future, more physiologically meaningful markers with which to determine the optimal timing of RRT initiation should be identified.

## Introduction

Acute kidney injury (AKI) is a common entity in critically ill patients with an incidence of about 30 to 60% [1] as defined by the RIFLE (risk, injury, failure, loss of kidney function, and end-stage renal failure) classification and is thought to be an independent risk factor for increased morbidity and mortality [2-4]. Sepsis is the leading cause of AKI, contributing to 30 to 50% of cases of AKI [4,5]. Almost 30% of septic AKI patients need renal replacement therapy (RRT). This rate is much higher than that observed for other causes of AKI [6-8]. Among critically ill patients, mortality rates of patients with septic AKI are also higher than among patients with non-septic AKI [9]. Thus, finding better strategies for septic AKI is the key issue for intensivists. The current goal is to improve strategies for the treatment of patients with septic AKI.

The pathogenesis of sepsis is a systemic inflammatory reaction that involves multiple inflammatory mediators. Many strategies for treatment were recommended as part of the early goal-directed therapy popularized by the Surviving Sepsis Campaign (SSC) [10]. Although RRT for refractory fluid overload, as well as electrolyte and acid-base imbalance, is recommended by the SSC, issues related to when and how to perform RRT are not addressed. Furthermore, continuous RRT (CRRT) with high-volume hemofiltration and a super-high flux dialyzer was suggested to restore immune homeostasis by removing cytokines and toxic molecules, but the effects on morbidity and mortality are still controversial [11,12].

As inflammatory cytokines play a critical role in the mechanism of septic AKI as compared with other etiologies of AKI [13], we hypothesized that the timing of RRT initiation in septic AKI is more important than in other types of AKI. However, certain observational studies showed that early initiation of RRT may be better for critically ill patients with severe AKI [14,15]. There is still no strong evidence or clear definition of how early is early enough. However, the RIFLE classification was used widely to categorize the severity of AKI, and was able to predict patient outcomes in some studies [16]. The purpose of the current study is to test the hypothesis that the timing of RRT initiation, as defined using sRIFLE criteria, is associated with patient outcomes, using our NSARF (National Taiwan University Hospital Study group on Acute Renal Failure) database.

## Materials and methods

### Study populations

This retrospective study was based on the NSARF database, which was established in the 64-bed surgical ICU of a tertiary hospital and its three branch hospitals in different cities [17-20]. The database prospectively collected data from patients requiring RRT during their ICU stays, and continuously recorded data from all patients for outcome analyses. In this study, we enrolled patients who underwent acute RRT because of septic AKI between July 2002 and October 2009. Those enrolled subjects were treated by one multi-modality team, composed of physicians, surgeons, technicians, and nursing personel. Septic AKI was defined as AKI development after sepsis without other etiology. Sepsis was classified according to the American College of Chest Physicians and the Society of Critical Care Medicine consensus [21]. Sepsis was defined by the presence of both infection and systemic inflammatory response syndrome (SIRS). SIRS was considered to: be present when patients had more than one of the following clinical findings: body temperature above 38°C or below 36°C, heart rate of more than 90 beats/min, hyperventilation evidenced by a respiratory rate of more than 20 breaths/min or a partial pressure of arterial carbon dioxide of less than 32 mmHg, and a white blood cell count of more than 12 × 10^3 ^cells/μl or less than 4 × 10^3 ^cells/μl. Infection was defined as a pathologic process caused by the invasion of normally sterile tissue or fluid or body cavity by pathogenic or potentially pathogenic microorganisms. Exclusion criteria included patients aged less than 18 years, patients with an ICU stay of less than two days [22], and patients who only underwent acute RRT for less than two days. Approval for this study was obtained from the Institutional Review Board of National Taiwan University Hospital, Taipei, Taiwan (No. 31MD03). Informed consent was waived because there was no breach of privacy and the study did not interfere with clinical decisions related to patient care.

### Data collection

All data were prospectively collected. Data variables included demographic data, comorbid diseases, septic AKI developed post-surgery (or not), and the indications for RRT. Biochemistry data such as complete blood cell count, blood urea nitrogen (BUN), serum creatinine (sCr), serum glutamate oxaloacetate transaminase (GOT), serum total bilirubin, serum albumin, and serum potassium (sK^+^) were recorded upon ICU admission and RRT initiation [18,20]. Moreover, the clinical parameters and severity score were also recorded at these two time points. The clinical parameters included heart rate, systolic and diastolic blood pressures, central venous pressure (CVP) level, partial pressure of arterial blood gas oxygen and fraction of inspired oxygen. Severity scores included Glasgow Coma Scale (GCS) score, Acute Physiology and Chronic Health Evaluation II (APACHE II) score [23], Sequential Organ Failure Assessment (SOFA) score [24], and Simplified Acute Physiology Score III (SAPS III) [25]. The usage of mechanical ventilation was recorded and the inotropic equivalent dose was calculated [26]. Definitions were made as follows: hypertension was blood pressure above 140/90 mmHg or usage of anti-hypertension agents; diabetes was previous usage of insulin or oral hypoglycemic agents; congestive heart failure was low cardiac output with a CVP above 12 mmHg and dopamine equivalent above 5 μg/kg/min [26]; and chronic kidney disease (CKD) was sCr of 1.5 mg/dl or greater documented prior to this admission.

The indications for RRT were: (1) azotemia (BUN > 80 mg/dL and sCr > 2 mg/dl) with uremic symptoms; (2) oliguria (urine amount <100 ml every eight hours) or anuria refractory to diuretics; (3) fluid overload refractory to diuretics with a CVP level above 12 mmHg or pulmonary edema with a partial pressure of arterial oxygen/fraction of inspired oxygen ratio below 300 mmHg; (4) hyperkalemia (sK^+ ^> 5.5 mmol/L) refractory to medical treatment; and (5) metabolic acidosis (pH <7.2 in arterial blood gas) [27].

According to previous studies [2,28,29], simplified RIFLE classification was used only with the glomerular filtration rate (GFR) criterion for classification because the eight-hourly urine volumes in our database did not match the 6- or 12-hourly urine output criterion in the RIFLE classification. The baseline sCr was the data acquired at hospital discharge from the prior admission among the patients with more than one admission [2], or the data estimated using the Modification of Diet in Renal Disease (MDRD) equation [30] in those with only one admission (assuming an average estimated GFR of 75 ml/min/1.73 m^2^). The peak sCr was defined as the highest sCr before RRT initiation in ICU. Those who initiated RRT when in sRIFLE-R (risk) or sRIFLE-0 [31], that is not yet reaching the sRIFLE-R level, were defined as the early dialysis (ED) group, while those in the sRIFLE-I (injury) or sRIFLE-F (failure) groups were classified as the late dialysis (LD) group.

### The choice of RRT modality

The modality of RRT was chosen according to the hemodynamics of the patients. Continuous venovenous hemofiltration was performed if more than 15 points of inotropic equivalent (IE) [26] were required to maintain systemic blood pressure up to 120 mmHg. The effluent flow and blood flow were 35 ml/kg/hour and 200 ml/min, respectively. Extended RRT such as sustained low efficiency RRT (SLED) with or without hemofiltration (SLED-f) was performed if IE was between 5 and 15 points. For SLED, blood flow and dialysate flow were 200 ml/min and 300 ml/min, respectively. When hemofiltration was added, the hemofiltration rate was 35 ml/kg/hour. The duration of hemofiltration was about 6 to 12 hours, according to the amount of ultrafiltration. Intermittent hemodialysis, which was chosen if IE was less than five points, was performed for four hours every session with a dialysate flow of 500 ml/min, and blood flow of 200 ml/min. As hemodynamics change, the patients may receive different RRT modalities [19].

### Outcomes

The endpoint of this study was in-hospital mortality. The survival period was calculated from RRT initiation to mortality (in non-survivors) or to hospital discharge (in survivors).

### Statistics

Statistical analyses were performed using SAS, version 9.1.3 (SAS Institute Inc., Cary, NC, USA), statistical software. In statistical testing, a two-sided *P *value of less than 0.05 was considered statistically significant. Continuous data were expressed as mean ± standard deviation unless otherwise specified. Frequency and percentage were calculated for categorical variables. Student's t test was used to compare the means of continuous data between two groups, whereas Chi-squared test or Fisher's exact test was used to analyze categorical proportions.

Then we used the backward stepwise likelihood ratio model of Cox proportional hazard method to analyze the independent predictors of in hospital mortality as model 1. The independent variables were selected for multivariate analysis if they had a *P *≤0.2 on univariate analysis. The basic model-fitting techniques for (1) variable selection, (2) goodness-of-fit assessment, and (3) regression diagnostics (e.g., residual analysis, detection of influential cases, and check for multicollinearity) were used in our regression analyzes to ensure the quality of the analysis results.

### Propensity matching

To balance the selection bias in an observational trial such as the current study, we used propensity score selection and the matching method [32,33]. Further models were adapted in our study. In model 2, we conducted Cox proportional hazard models using a propensity score, and included all patients based on the probability of late RRT. In model 3, we identified factors associated with late RRT in the entire cohort, using stepwise logistic regression. Based on the factors identified, we matched patients with 1:1, 2:2, 3:3, or 4:4 blocks manually [32]. We subsequently compared outcomes between patients undergoing early dialysis or late dialysis. In addition, a sensitivity analysis was also carried out among the subset of patients undergoing dialysis due to azotemia, which represented the largest proportion of our study population.

Finally, Kaplan-Meier curves obtained with the log-rank test were plotted to demonstrate the differences in patient survival between the two groups (ED versus LD).

## Results

From our database, we identified 1,258 patients who underwent RRT during the study period. Among these patients, 370 fulfilled our enrollment and exclusion criteria for septic AKI. The mean age of enrolled patients was 65.4 ± 15.9 years on the day of RRT. Males accounted for 67.0% of patients. The basic demographic data on enrollment and on ICU admission and acute physiology scores are shown in the upper part of Table 1. Finally, 192 (51.9%) patients underwent early RRT and the rest (48.1%) received late RRT. In-hospital mortality affected 279 patients (70%). Hospital mortality rates were comparable in these two groups (70.8% vs. 69.7%, respectively; *P *= 0.98).

**Table 1:** Comparisons of demographic data and clinical parameters among the whole cohort as well as early, and late RRT groups (*n *= 370)

	Enrolled patients (*n *= 370)	Early RRT (*n *= 192)	Late RRT (*n *= 178)	*P*-value
**Demographic data**				
Age (years)	65.4 ± 15.9	64.1 ± 16.5	66.7 ± 15.2	0.34
Male (%)	248 (67.0)	119 (62)	129 (72.5)	0.04
DM (%)	126 (34.1)	63 (32.8)	63 (35.5)	0.68
Hypertension(%)	175 (47.3)	92 (47.9)	83 (46.6)	0.93
CHF (%)	68 (18.4)	44 (22.9)	24 (13.5)	0.02
CKD (%)	92 (24.9)	56 (29.2)	36 (20.2)	0.06
Post-operative (%)	237 (64.1)	113 (58.9)	124 (69.7)	0.03
Admission creatinine (mg/dL)	2.0 ± 0.2	2.7 ± 1.7	1.5 ± 1.0	< 0.01
(μmol/L)	176.6 ± 17.7	238.7 ± 150.3	132.6 ± 88.4	
Mechanical ventilation (%)	322 (87.0)	163 (84.9)	159 (89.3)	0.27
**Data at ICU admission**				
Hematocrit (%)	32.2 ± 9.5	32.3 ± 12.0	32.1 ± 5.9	0.83
BUN (mg/dL)	50.7 ± 33.7	59.8 ± 34.1,	41.0 ± 30.4,	0.05
(mmol/L)	18.1 ± 12.0	21.3 ± 12.2	14.6 ± 10.9	
Creatinine (mg/dL)	2.5 ± 1.7	2.6 ± 1.8	2.3 ± 1.6	0.06
(μ mol/L)	221 ± 150.3	232.5 ± 156.5	200.7 ± 141.4	
Albumin (g/dL)	2.9 ± 0.7	3,0 ± 0.7	2.9 ± 0.7	0.08
(g/L)	29 ± 7	30 ± 7	29 ± 7	
APACHE II scores	11.3 ± 6.4	11.8 ± 6.6	10.7 ± 6.0	0.37
SOFA scores	8.1 ± 3.8	8.7 ± 3.7	7.4 ± 3.8	0.18
SAPS III score	63.1 ± 13.0	64.7 ± 7.3	62.2 ± 7.6	0.30
**Pre-RRT data**				
Hematocrit (%)	30.0 ± 5.8	30.1 ± 6.3	29.8 ± 0.6	0.62
BUN (mg/dL)	81 ± 40.6	78.2 ± 41.2	84.0 ± 39.8	0.19
(mmol/L)	28.9 ± 14.5	27.9 ± 14.7	30.0 ± 14.2	
Creatinine (mg/dL)	3.4 ± 0.4	3.4 ± 0.3	3.4 ± 0.8	0.17
(μmol/L)	298.8 ± 35.4	297.9 ± 23.9	299.7 ± 70.7	
Albumin (g/dL)	3.0 ± 1.2	3.1 ± 1.5	2.9 ± 0.6	0.09
(g/L)	30 ± 12	31 ± 15	29 ± 6	
Potassium (mEq/L)	4.8 ± 12.1	5.0 ± 1.0	4.3 ± 0.9	0.32
Lactate (mg/dL)	3.4 ± 3.6	3.5 ± 3.9	3.1 ± 3.5	0.10
(mmol/L)	0.4 ± 0.4	0.4 ± 0.4	0.3 ± 0.4	
GCS scores	11.8 ± 3.7	11.5 ± 4.3	11.0 ± 4.5	0.72
Systolic blood pressure	122.7 ± 25.7	122.8 ± 26.5	123.7 ± 25.7	0.84
Diastolic blood pressure	61.2 ± 39.0	61.5 ± 14.1	60.1 ± 14.0	0.36
Central venous pressure	13.8 ± 5.5	14.16 ± 5.9	13.4 ± 5.2	0.22
APACHE II scores	13.1 ± 6.4	12.3 ± 7.0	14.0 ± 5.5	0.52
SOFA scores	11.2 ± 3.9	10.8 ± 4.0	11.6 ± 3.7	0.80
SAPS III score	67.3 ± 6.8	66.2 ± 6.7	68.6 ± 6.7	0.61
**Indications for dialysis**				
Azotemia with uremic symptoms	265 (71.6)	119 (62.0)	146 (82.0)	< 0.01
Oligouria or anuria	241 (65.1)	113 (58.9)	128 (63.0)	0.01
Fluid overload	81 (21.9)	41 (21.4)	40 (22.5)	0.29
Electrolyte imbalance	23 (6.2)	8 (4.2)	15 (8.4)	0.14
Acid base imbalance	25 (6.8)	12 (6.4)	13 (7.3)	0.84
Rhabdomyolysis	7 (1.9)	5 (2.7)	2 (1.1)	0.51
**Hospital mortality**	259 (70.0)	135 (70.8)	124 (69.7)	0.98

Values are presented as mean ± standard deviation or number (percentage) unless stated otherwise.

APACHE II: Acute Physiology and Chronic Health Evaluation II; BUN: blood, urea, nitrogen; CHF: congestive heart failure; CKD: chronic kidney disease; DM: diabetes mellitus; GCS: Glasgow Coma Scale; RRT: renal replacement therapy; SAPS: Simplified Acute Physiology Score; SOFA: Sequential Organ Failure Assessment.

### Model 1: general model (Table 2)

**Table 2:** Independent predictors of in-hospital mortality obtained using the Cox proportional hazards model

Variables	Unadjusted (model 1)			Propensity score adjusted (model 2)		
	HR	95% CI	*P*-value	HR	95% CI	*P*-value
Post-operative, yes	0.631	0.478 - 0.832	0.0011			
Pre-RRT CVP (mmHg)	1.030	1.006 - 1.055	0.0140			
Pre-RRT DBP (mmHg)	0.987	0.977 - 0.997	0.0089	0.987	0.977 - 0.997	0.013
Pre-RRT GCS scores	0.929	0.898 - 0.962	< 0.001	0.923	0.890 - 0.958	< 0.001
Pre-RRT lactate (mM)	1.086	1.048 - 1.124	< 0.001	1.073	1.034 - 1.113	< 0.001
SOFA score on ICU admission	0.941	0.907 - 0.977	0.0015	0.934	0.900 - 0.970	< 0.001
SOFA score pre-RRT	1.068	1.019 - 1.120	0.0058	1.104	1.051 - 1.160	< 0.001
Propensity scores	-	-	-	0.085	0.027 - 0.268	0.085

95% CI: 95% confidence interval; CVP: central venous pressure; DBP: diastolic blood pressure; GCS: Glasgow Coma Scale; HR: hazard ratio; RRT: renal replacement therapy; SOFA: Sequential Organ Failure Assessment.

Cox proportional hazard model were conducted with the whole cohort to identify factors associated with in-hospital mortality. We found that patients underwent operations before RRT (hazard ratio (HR) = 0.631, *P *= 0.0011), pre-RRT CVP (HR = 1.030, *P *= 0.0140), pre-RRT diastolic blood pressure (HR = 0.987, *P *= 0.0089), pre-RRT GCS scores (HR = 0.929, *P *< 0.001), pre-RRT plasma lactate (mM) (HR = 1.086, *P *< 0.001), SOFA score on ICU admission (HR = 0.941, *P *= 0.0015), and SOFA scores on RRT commencement (HR = 1.068, *P *= 0.0058) were independently associated with in-hospital mortality.

### Model 2: propensity score adjusted methods (Table 2)

The Cox proportional hazard model was conducted using the whole cohort, including propensity score as a covariate, and identified pre-RRT diastolic blood pressure (HR = 0.987, *P *= 0.013), pre-RRT GCS scores (HR = 0.923, *P *< 0.001), pre-RRT lactate (mM) (HR = 1.073, *P *< 0.001), pre-RRT SOFA score (HR = 1.104, *P *< 0.001), and SOFA score on ICU admission (HR = 0.934, *P *< 0.001) predicted in-hospital mortality when propensity scores were conditioned (HR = 0.085, *P *< 0.001).

### Model 3: propensity score matching method

By logistic regression, we identified differences between early RRT and late RRT groups, and found that male patients (odds ratio (OR) = 1.588, 95% confidence interval (CI) = 1.012 to 2.492, *P *= 0.0444), patients with CHF (OR = 0.520, 95% CI = 0.297 to 0.909, *P *= 0.0217), patients receiving operations (OR = 1.618, 95% CI = 1.041 to 2.516, *P *= 0.0326), and patients with higher admission creatinine (OR = 1.184, 95% CI = 1.051 to 1.333, *P *= 0.0055) could predicted late dialysis. This model had a good discriminating power (c-index = 0.637), and validation (Hosmer-Lemshow's statistics, *P *= 0.07, with chi squared = 14.6, df = 8) was fair.

**Figure 1:**
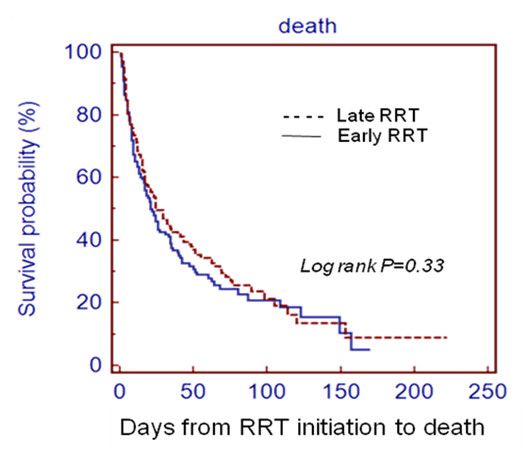
**Comparison of cumulative patient survival between early and late dialysis groups, as defined by the sRIFLE classification**. By Kaplan-Meier method. Dashed line, late dialysis group (sRIFLE-I and sRIFLE-F). Solid line, early dialysis group (sRIFLE-0 and sRIFLE-R). RRT: renal replacement therapy.

We matched patients by 1:1 fashion according to each patient's propensity to late RRT. After careful matching, there were 178 patients in each cohort. Table 3 showed the demographic data of the matched cohort. No differences about hospital mortality were detected in both groups according to head-to-head comparison of demographic data. Log Rank test of Kaplan-Meier curves (Figure 1) was insignificant between these two groups (HR = 1.13, *P *= 0.33).

**Table 3:** Comparisons of demographic data and clinical parameters between matched early, and late RRT groups (model 3)

	Early RRT (*n *= 178)	Late RRT (*n *= 178)	*P*-value
**Demographic data**			
Age (years)	65.2 ± 15.6	66.7 ± 15.2	0.71
Male (%)	63.5	72.5	0.07
DM (%)	32.7	35.5	0.71
Hypertension (%)	47.8	46.6	0.83
CHF (%)	17.4	13.5	0.38
CKD (%)	28.1	20.2	0.06
Post-operative(%)	62.9	69.7	0.21
Admission creatinine (mg/dL)	1.9 ± 1.1	1.5 ± 1.0	0.07
(mmol/L)	168.0 ± 97.2	132.6 ± 88.4	
Mechanical ventilation(%)	85.4	89.3	0.34
**Data at ICU admission**			
Hematocrit (%)	32.2 ± 12.1	32.1 ± 5.9	0.17
BUN (mg/dL)	50.8 ± 34.4	41.0 ± 30.4	0.07
(mmol/L)	18.1 ± 12.2	14.6 ± 10.9	
Creatinine (mg/dL)	2.9 ± 1.8	2.3 ± 1.6	0.13
(μmol/L)	247.5 ± 159.1	200.7 ± 141.4	
Albumin (g/dL)	2.9 ± 0.6	2.9 ± 0.7	0.13
(g/L)	29 ± 6	29 ± 7	
APACHE II scores	11.9 ± 6.8	10.7 ± 6.0	0.83
SOFA scores	8.9 ± 3.7	7.4 ± 3.8	0.61
SAPS III score	60.7 ± 12.3	62.2 ± 7.6	0.25
**Pre-RRT data**			
Hematocrit (%)	30.0 ± 6.2	29.8 ± 0.6	0.19
BUN (mg/dL)	79.1 ± 41.4	84.0 ± 39.8	0.26
(mmol/L)	28.3 ± 14.8	30.0 ± 14.2	
Creatinine (mg/dL)	3.4 ± 1.9,	3.4 ± 0.8	0.25
(μmol/L)	300.6 ± 168.0	299.7 ± 70.7	
Albumin (g/dL)	3.1 ± 1.5	2.9 ± 0.6	0.10
(g/L)	31 ± 15	29 ± 6	
Potassium (mEq/L)	5.1 ± 1.0	4.3 ± 0.9	0.16
Lactate (mg/dL)	3.4 ± 3.6	3.1 ± 3.5	0.14
(mmol/L)	0.4 ± 0.4	0.3 ± 0.4	
GCS scores	11.7 ± 4.2	11.0 ± 4.5	0.40
Systolic blood pressure	123.5 ± 26.7	123.7 ± 25.7	0.37
Diastolic blood pressure	64.6 ± 54.3	60.1 ± 14.0	0.35
Central venous pressure	14.3 ± 5.7	13.4 ± 5.2	0.85
APACHE II scores	12.3 ± 6.7	14.0 ± 5.5	0.34
SOFA scores	10.9 ± 4.1	11.6 ± 3.7	0.13
SAPS III score	66.3 ± 6.8	68.6 ± 6.7	0.79
**Hospital mortality **(%)	70.8	69.7	0.98

Values are presented as mean ± standard deviation or number (percentage) unless otherwise stated.

APACHE II: Acute Physiology and Chronic Health Evaluation II; BUN: blood, urea, nitrogen; CHF: congestive heart failure; CKD: chronic kidney disease; DM: diabetes mellitus; GCS: Glasgow Coma Scale; RRT: renal replacement therapy; SAPS: Simplified Acute Physiology Score; SOFA: Sequential Organ Failure Assessment.

Further sensitivity analyzes were undertaken using patients undergoing RRT because of uremic symptoms. Hospital mortality was associated with post-operative status (HR = 0.651, *P *= 0.002), pre-RRT CVP level (HR = 1.031, *P *= 0.002), pre-RRT diastolic blood pressure (HR = 0.9687, *P *= 0.0029), pre-RRT GCS scores (HR = 0.969, *P *< 0.0001), pre-RRT lactate level (HR = 1.091, *P *< 0.0001), SOFA score on ICU admission (HR = 0.921, *P *= 0.0033), and SOFA score on starting RRT (HR = 1.071, *P *= 0.0021).

## Discussion

Whether or not to perform and when to start RRT in patients with AKI are two dilemmas facing intensivists. There is still no consensus and the initiation of RRT is extremely variable and based primarily on empiricism, local institutional practice, and resources [5,34,35]. Traditional indications for RRT among end-stage renal disease patients were not appropriate for AKI patients. The concepts of renal support for AKI patients were established in 2001 by Mehta [36]. Some indicators for RRT and renal support are the same in life-threatening conditions such as severe hyperkalemia, marked acid-base disturbances, or diuretic-resistant pulmonary edema. Other indications may differ between patients with end-stage renal disease and AKI. For instance, many studies have found that even mild increases in sCr in AKI patients have significant impact on outcome [37]. Interventions should be performed earlier and dialysis may be considered if residual renal function cannot support the patient.

As patients who received dialysis too early would be exposed to unnecessary risk of dialysis, while those exposed too late may experience worse outcomes, establishing reliable indications for RRT in patients with AKI is important. In many studies, BUN and sCr are used to categorize early and late dialysis patients. This topic has been debated since the 1960s [38]. The first result of improved survival rates in patients dialyzed with a lower starting BUN was published by Teschan *et al *[39]. Regarding recent studies, some retrospective studies reported better survival among patients with post-traumatic and post-operation AKI who received early RRT. [40-42]. However, other studies found no difference in survival in the early-dialyzed group of critically ill patients [14,15,43]. It is difficult to compare or perform meta-analysis among the aforementioned studies due to the lack of consensus on the definitions of early and late RRT. Due to extensive validation of the RIFLE classification, some studies have used it to evaluate the relation between prognosis and RRT timing [28,42].

Our study focused on the impact of timing for septic AKI because of the unique pathogenesis of septic AKI, which involves a deleterious inflammatory cascade mediated by cytokines and toxic molecules. Some studies have demonstrated improvement in hemodynamics and mortality by RRT [13]. However, there are very few reports addressing the impact of RRT timing on septic AKI. A study by Liu *et al*. [15] included numerous patients who had sepsis or septic shock (37% in the early group and 46% in the late group) and is the first study to address the timing of RRT in critically ill septic patients. Also, the most common cause of AKI in the study by Bagshaw *et al*. was septic shock [9]. However, the study was not specifically designed to look at timing of RRT in critically ill patients with sepsis, and their definitions of timing were different. Our previous study found that late initiation of RRT was associated with worse outcomes in AKI after major abdominal surgery [42]. The patients were divided to ED (simplified RIFLE-0 or Risk) and LD (simplified RIFLE-Injury or Failure) groups; 27.5% and 36.2% patients in the early and late RRT groups, respectively, had sepsis. Carl *et al*. retrospectively reviewed the effect of timing of RRT on mortality among critically ill, septic patients with AKI [44]. They found survival rates for the ED group were significantly higher than that in the LD group.

Although we matched early and late RRT groups with propensity score, there was still no survival benefit in the early RRT group. One possibility is that our patients may receive different modalities, depending on their hemodynamics. Therefore, there was no fixed dose or modality during RRT that influenced the outcome. Another reason may be that traditional markers were not sufficiently sensitive to detect AKI early on. For example, Doi *et al*. found that creatinine production is reduced in sepsis [45]. As Gibney *et al*. and Macedo *et al*. recently reported, it is reasonable to combine other independent predictors of varying weight to calculate an index for use by intensivists in determining the optimal timing of RRT initiation [46,47].

In the propensity score adjusted model, we found some predictors of in-hospital mortality among septic AKI patients (Table 2). Lower pre-RRT diastolic blood pressure, lower pre-RRT GCS scores, and higher pre-RRT lactate level may be due to poor heart function and poor tissue perfusion, which resulted in poor outcome [14,48]. In addition, higher ICU admission and pre-RRT SOFA score predicted a higher risk of death. The SOFA score has been reported to be a prognostic factor in other studies [29,49].

### Limitations and summary

As an observational study, this investigation has several limitations to be addressed. First, only the estimated GFR criterion of the RIFLE classification was used in this study. Urine output may also be an important indication for initiation of RRT. Thus we used the term 'sRIFLE' to distinguish this classification from the original RIFLE. Second, our patients received different modalities as dictated by their individual hemodynamics. Therefore, we were not able to manage the impact of different doses and modalities. Third, we only included patients who actually underwent RRT. There will be a subset of patients with AKI who are not dialyzed "early" who never undergo RRT because they either die or recover kidney function before meeting the "late" criteria for RRT. Fourth, we did not correct sCr by the degree of fluid [50].

## Conclusions

Patients with AKI associated with sepsis carry substantial risks for adverse outcomes, especially those who need RRT. Timely RRT has been proposed as an attractive modality to improve patient outcomes in septic AKI patients; but our current analyzes did not support early RRT, as defined with sRIFLE classification. Future research efforts should seek to identify more physiologically meaningful markers to determine the optimal timing of RRT initiation.

## Key messages

• Timely RRT initiation in patients with septic AKI is important, but previous studies did not demonstrate strong evidence or clear definition of how early is early enough.

• Pre-RRT diastolic blood pressure, pre-RRT GCS scores, pre-RRT lactate level, pre-RRT SOFA score, and SOFA score on ICU admission predicted in-hospital mortality.

• Early or late RRT, as defined with simplified RIFLE classification, could not predict in-hospital mortality.

• More physiologically meaningful markers with which to determine the optimal timing of RRT initiation should be identified.

## Abbreviations

AKI: acute kidney injury; APACHE II: Acute Physiology and Chronic Health Evaluation II; BUN: blood urea nitrogen; CI: confidence interval; CKD: chronic kidney disease; CRRT: continuous renal replacement therapy; CVP: central venous pressure; ED: early dialysis; GCS: Glascow Coma Scale; GFR: glomerular filtration rate; GOT: glutamate oxaloacetate transaminase; HR: hazard ratio; IE: inotropic equivalent; LD: late dialysis; MDRD: Modification of Diet in Renal Disease; OR: odds ratio; RIFLE: risk, injury, failure, loss of kidney function, and end-stage renal failure; RRT: renal replacement therapy; SAPS: Simplified Acute Physiology Score; sCr: serum creatinine; SIRS: systemic inflammatory response syndrome; sK^+^: serum potassium; SLED: sustained low efficiency dialysis; SOFA: Sequential Organ Failure Assessment; sRIFLE: simplified RIFLE (risk, injury, failure, loss of kidney function, and end-stage renal failure) classification; SSC: Surviving Sepsis Campaign.

## Competing interests

The authors declare that they have no competing interests.

## Authors' contributions

YHC conceived the study and participated in data collection and manuscript writing. TMH performed statistical analysis and manuscript writing. VCW, CYW, CCS, YFL, and YMC participated in data collection and manuscript revision. PRT, YYH, AC, THL, YWY, and NKC participated in data collection. WJK participated in data collection and manuscript writing. KDW conceived the study and participated in manuscript revision. CFL, HBT, CTC, GHY, WJW, TWK, JWH, WCC, TJT, and SLL participated in manuscript revision. All authors read and approved the final manuscript.
